# Performance of overnight on-call radiology residents in interpreting unenhanced abdominopelvic magnetic resonance imaging studies performed for pediatric right lower quadrant abdominal pain

**DOI:** 10.1007/s00247-021-05009-8

**Published:** 2021-03-10

**Authors:** David M. Sawyer, Raza Mushtaq, Srinivasan Vedantham, Faryal Shareef, Sara M. Desoky, Hina Arif-Tiwari, Dorothy L. Gilbertson-Dahdal, Unni K. Udayasankar

**Affiliations:** 1 Department of Medical Imaging, University of Arizona, 1501 N. Campbell, P.O. Box 245067, Tucson, AZ 85724 USA; 2 Creighton University of Arizona Health Alliance, Phoenix, AZ USA

**Keywords:** Abdomen, Appendicitis, Children, Magnetic resonance imaging, Pain, Preliminary report, Trainees

## Abstract

**Background:**

Abdominopelvic magnetic resonance imaging (MRI) is increasingly being used to evaluate children with abdominal pain suspected of having acute appendicitis. At our institution, these examinations are preliminarily interpreted by radiology residents, especially when performed after hours.

**Objective:**

To determine the accuracy of preliminary reports rendered by radiology residents in this setting.

**Materials and methods:**

Three hundred seventy-seven pediatric abdominopelvic MRI examinations were included. The preliminary (resident) and final (attending) radiology reports were coded as diagnosing acute appendicitis or no acute appendicitis. The concordance between resident and attending radiologist interpretations was calculated. Additionally, both resident and attending reports were compared to available surgical pathology or clinical follow-up data.

**Results:**

Overall concordance rate for the diagnosis of acute appendicitis was 97.1%. Concordance for verified cases of acute appendicitis was 93.4%. Concordance rates did not differ by residents’ postgraduate year levels. When compared against surgical pathology or clinical follow-up data, residents demonstrated 91.2% sensitivity and 97.6% specificity. There was no statistically significant difference in the sensitivity or specificity of resident or attending radiologist interpretations.

**Conclusion:**

Radiology residents demonstrate high concordance with attending pediatric radiologists in their interpretations of pediatric abdominopelvic MRI for acute appendicitis. The diagnostic performances of residents and attendings were comparable.

## Introduction

Acute appendicitis represents a prevalent and important cause of abdominal pain in pediatric patients [1]. Rapid diagnosis and identification of complications are vital for timely and appropriate management of this condition. Imaging plays a crucial role in evaluating children with suspected acute appendicitis and has been shown to reduce negative appendectomy rates [2].

Although there is no universally accepted strategy for imaging pediatric patients with suspected acute appendicitis, some guidance is available. The American College of Radiology (ACR) has recently published updated appropriateness criteria on this topic [3]. These guidelines recommend initial evaluation with ultrasound (US) for patients with an intermediate risk of acute appendicitis based on clinical assessment, followed by further evaluation with computed tomography (CT) or magnetic resonance imaging (MRI) in cases with equivocal US findings. In the setting of a high clinical risk, the three modalities carry equivalent recommendations of “may be appropriate,” while all three are “usually not appropriate” in the setting of a low clinical risk.

Ultrasound has clear benefits in the setting of pediatric right lower quadrant abdominal pain, including its lack of ionizing radiation and intravenous contrast, low cost and availability. It can achieve very high sensitivity and specificity for acute appendicitis under ideal conditions [4]. However, the accuracy of US varies with operator skill, patient factors and clinical setting [3] and the 2018 ACR Appropriateness Criteria make the assumption of US performance by an expert when making its recommendations. CT is more likely to be the initial diagnostic modality in a community hospital setting [5]. The benefits and risks of CT in the diagnosis of pediatric appendicitis have been well characterized; while CT offers high diagnostic accuracy [6, 7], concerns regarding radiation exposure limit its utility as a first-line test in the pediatric population.

There has been rising interest in MRI for pediatric appendicitis. Diagnostic accuracy of the modality is high [8], but its utility was shown in some studies to be limited by long imaging times (often requiring sedation in children) and the need for gadolinium-based intravenous contrast administration. However, advances in MRI protocols have allowed for rapid non-contrast examinations that maintain a high degree of sensitivity/specificity [9, 10]. A recent study demonstrated the efficacy of MRI when employed as a first-line modality in pediatric patients suspected of having acute appendicitis [11].

However, there is a question of generalizability of these results. Given that the majority of studies published on MRI have relied on the interpretations of board-certified pediatric radiologists, it remains to be shown that the modality could effectively be employed by trainees or general/emergency radiologists in the community setting. The purpose of this study is to evaluate the diagnostic performance of radiology residents in preliminarily interpreting first-line MRI studies performed in children with clinical concern for acute appendicitis.

## Materials and methods

This retrospective chart review study was approved by our institutional review board, with a waiver of informed consent.

### Clinical setting

At our institution, pediatric acute abdomen MRI performed outside of normal business hours is supervised and interpreted by second-year (PGY-3) and above diagnostic radiology residents with indirect attending pediatric radiologist supervision. Pediatric radiology attendings are available in-house from 8 a.m. to 5 p.m.; however, during these hours, residents often render preliminary reports for these studies before an official readout with the attending. After hours, the on-call attending is not physically present in-house but is immediately available by phone and able to review studies from home if necessary. In general, MRI examinations are performed according to a standardized protocol and completed by the technologist without real-time radiologist supervision. Further detail regarding workflow in our department, including average times from MRI order to image acquisition and interpretation, can be found in a recently published study [11].

### Data collection

A list of consecutive patients 21 years old or younger (the cut-off age for our pediatric emergency department) who presented to our institution with acute abdominal pain between January 2013 and June 2016 and underwent an unenhanced MRI examination of the abdomen and pelvis was generated from the hospital electronic medical records. During this time period, our institution implemented a cross-departmental strategy of performing MRI as the initial imaging modality in cases of suspected acute appendicitis in pediatric patients. As many patients as possible were imaged using MRI as the first-line test, and patients as young as 3 years old were successfully examined.

A number of patients from the initial list were excluded from the study. Exclusion criteria included: (1) age 18 years or older (in order to better adhere to the commonly held definition of a pediatric cohort), (2) MRI following either a CT or US examination, (3) incomplete MRI, (4) previous history of appendectomy and (5) lost to follow-up (defined by a lack of subsequent visits recorded in the electronic medical record). This cohort of patients has been examined in a previous study [11]; additional exclusion criteria were applied in the current study, such that the 377 patients included in the current study overlap with the 402 patients in the previous study. The additional exclusion criteria for the current study were patients whose reports were rendered exclusively by an attending radiologist or generated by a first-year resident under direct attending supervision. These additional exclusion criteria were used to exclude cases in which the preliminary resident report was not rendered by an “independent” resident.

### Magnetic resonance imaging protocol

All MRI examinations were performed on one of two available scanners, a 1.5 tesla (T) Magnetom Aera or a 3.0-T Magnetom Skyra (Siemens Healthcare, Erlangen, Germany). Multisequence, multiplanar imaging was performed without gadolinium-based contrast. The protocol, which was standardized for all patients, included the following sequences: T2-weighted single-shot fast spin echo (axial, coronal and sagittal planes), fat-suppressed T2-weighted single-shot fast spin echo (axial, coronal and sagittal planes), T1-weighted three-dimensional (3-D) dual-echo spoiled gradient recalled echo (axial plane), diffusion-weighted imaging (axial plane), and 3-D T2-weighted turbo spin echo (axial plane, pelvis only). A complete technical description of imaging parameters has been previously published [11]. Moderate sedation was administered by the treating emergency department physician in rare cases when deemed clinically necessary. A small minority of cases were performed under general anesthesia.

### Magnetic resonance imaging interpretation

The imaging criteria used for acute appendicitis in this retrospective study were not strictly standardized and diagnosis was made at the discretion of each reader. However, commonly reported MRI findings indicative of acute appendicitis included a fluid-filled appendix, appendiceal wall edema, periappendiceal inflammatory changes and an appendicolith. For the purposes of this study, cases of complicated appendicitis (perforation, abscess) were not specifically delineated and were simply included as cases of positive acute appendicitis.

### Reference standards

The electronic medical record for each patient was reviewed to serve as the reference standard for acute appendicitis (true positive or true negative). For patients who underwent surgery, surgical histopathological findings served as the reference standard. For patients who did not undergo surgery, follow-up clinical evaluations documenting favorable response to conservative management served as the reference standard. These evaluations occurred in the form of follow-up to the patient’s emergency department visit or as part of the patient’s next well-child visit.

### Concordance and diagnostic performance

The preliminary (resident) and final (attending) interpretations of each MRI examination were reviewed. Interpretations were categorized as either positive or negative for acute appendicitis. For the purposes of this study, only interpretations that were unequivocally negative (e.g., reports stating “negative for acute appendicitis,” “no evidence of acute appendicitis” or “normal appendix”) were categorized as negative. Reports that indicated a low but nonzero degree of suspicion for acute appendicitis (e.g., “equivocal for acute appendicitis” or “could represent early appendicitis”) were categorized as a positive interpretation.

Concordance between resident and attending reports was determined. Additionally, diagnostic performance (sensitivity, specificity, and positive and negative predictive value) of both residents and attending radiologists was calculated by comparing the resident and attending reports to the reference standards described above.

### Alternative diagnoses

The studies in the data set that were negative for acute appendicitis were reviewed to determine whether an alternative diagnosis explaining the patient’s abdominal pain was identified. In cases where an alternative diagnosis was identified in the attending report, a comparison was made between the resident and attending reports to determine concordance between the resident and attending radiologists.

### Statistical methods

Categorical variables were numerically coded. Continuous variables were tested for normality assumption (Shapiro-Wilk W test) and appropriate summary statistics were reported. For the paired interpretations by the residents and the attending radiologists, McNemar’s test of correlated proportion was used to determine if the positive interpretation rate for acute appendicitis differed. Fisher exact tests were used to determine if the concordance rates differed with the resident’s level of training and with the academic year. For the metrics reported as a proportion or a ratio, the exact (Clopper-Pearson) 95% confidence intervals were obtained using simple binomial proportions. All analyses were performed using statistical software (SAS version 9.4; SAS Institute Inc., Cary, NC). Effects associated with *P*<0.05 were considered statistically significant.

## Results

**Fig. 1:**
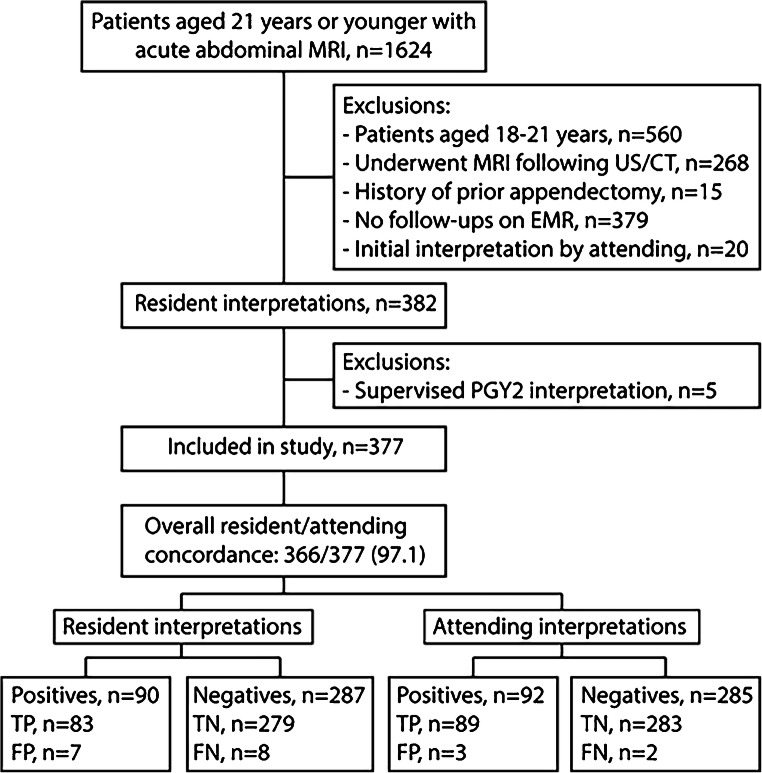
Study flowchart. *EMR* electronic medical records, *FN* false negatives, *FP* false positives, *PGY2* post-graduate year 2, *TN* true negatives, *TP* true positives

A total of 377 patients were included in this study; 231 patients were scanned after hours (62.3%). A flowchart describing the process of data collection can be seen in Fig. 1. The demographic and clinical characteristics of the included patients are summarized in Table 1.

**Table 1:** Summary statistics of patient characteristics

Characteristic	Data
Total number of patients	377
Females	223 (59.2%)
Males	154 (40.8%)
Age (y)^a^	13 (9–15)
Age of females (y)^a^	14 (10–15)
Age of males (y)^a^	11 (8–15)
Presenting symptoms	
RLQ abdominal pain	253/377 (67.1%)
Nonspecific abdominal pain	156/377 (41.4%)
Vomiting	197/377 (52.3%)
Fever	126/377 (33.4%)
Other symptoms	170/377 (45.1%)
White blood cell count (10^9^ per liter)^a^	10.9 (7.9–15.4)
Number of patients sedated	12/377 (3.2%)

*RLQ* right lower quadrant, *y* years

^a^Reported as median (interquartile range)

### Concordance

**Fig. 2:**
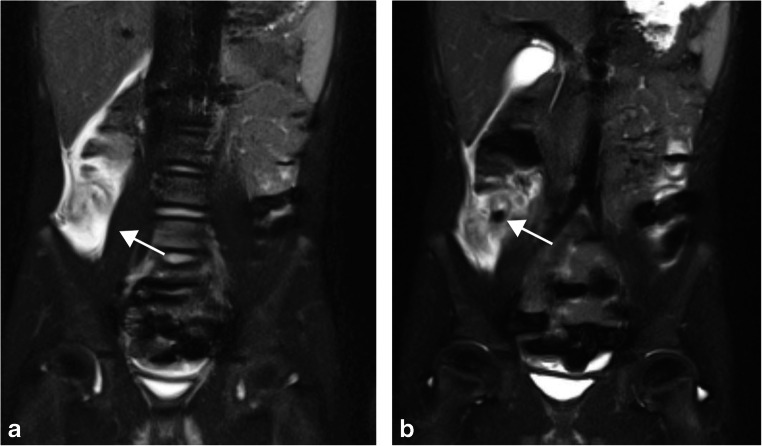
A 6-year-old boy with acute appendicitis. **a**, **b** Coronal fat-suppressed T2-weighted single-shot fast spin echo images demonstrate an enlarged, fluid-filled appendix with significant surrounding inflammatory changes (*arrow* in **a**). An appendicolith (*arrow* in **b**) is at the base of the appendix. Acute appendicitis was confirmed at surgery and histology

Of the 377 patients included in the study, there were 91 positive cases of acute appendicitis and 286 negative cases. A representative positive case can be seen in Fig. 2. Initial interpretations were provided by second-year residents in 214 cases, third-year residents in 99 cases and fourth-year residents in 64 cases. The concordance rate between resident interpretations and attending radiologist interpretations is summarized in Table 2. For all patients, the overall concordance rate across all resident training levels was 97.1% (366/377). The concordance rate between the attending radiologist and the resident did not differ significantly with the resident’s training level (*P*=0.28, Fisher exact test) and showed overlapping 95% confidence intervals (Table 2). The concordance rate also did not differ significantly among the four academic years that encompass the study time period (*P*=0.24, Fisher exact test) and demonstrated overlapping 95% confidence intervals (Table 3).

**Table 2:** Concordance rates between resident interpretations and attending radiologist interpretations, by resident training level

Resident training level	All patients	Patients with verified diagnosis of appendicitis
All training levels	366/377, 97.1% (94.8–98.5%)	85/91, 93.4% (86.2–97.5%)
Second year	210/214, 98.1% (95.3–99.5%)	47/50, 94.0% (83.5–98.8%)
Third year	95/99, 96.0% (90.0–98.9%)	21/23, 91.3% (72.0–98.9%)
Fourth year	61/64, 95.3% (86.9–99.0%)	17/18, 94.4% (72.7–99.9%)

Numbers in parentheses indicate 95% confidence intervals

**Table 3:** Concordance rates between resident interpretations and attending radiologist interpretations for various academic years

Academic year	All patients	Patients with verified diagnosis of appendicitis
2012–2013	45/48, 93.8% (82.8–98.7%)	10/12, 83.3% (51.6–97.9%)
2013–2014	149/151, 98.7% (95.3–99.8%)	37/37, 100% (90.5–100%)
2014–2015	160/166, 96.4% (92.3–98.7%)	35/39, 89.7% (75.8–97.1%)
2015–2016	12/12, 100% (73.5–100%)	3/3, 100% (29.2–100%)

Numbers in parentheses indicate 95% confidence intervals

For the 91 patients with verified diagnosis of appendicitis, the overall concordance rate across all resident training levels was 93.4% (85/91), did not differ significantly with the resident’s training level (*P*=0.86, Fisher exact test) and demonstrated overlapping 95% confidence intervals (Table 2). Also, the concordance rate did not differ significantly over the four academic years (*P*=0.09, Fisher exact test).

**Fig. 3:**
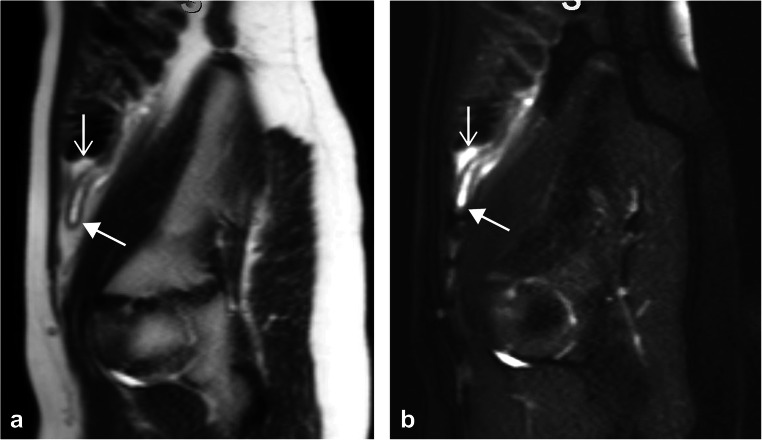
A 9-year-old boy with subtle acute appendicitis. **a**, **b** Sagittal T2-weighted single-shot fast spin echo images without (**a**) and with (**b**) fat suppression. The appendix is mildly dilated and fluid-filled with mild wall thickening (*lower arrows*). There is a small amount of periappendiceal inflammatory edema (*upper arrows*). This case was originally interpreted as negative for acute appendicitis by the resident. The attending interpretation indicated acute appendicitis, which was confirmed at surgery and histology

There were 11 cases of discrepant interpretations in the data set. A representative discrepant case is presented in Fig. 3. We subjectively examined these cases in more detail to better understand the nature of these discrepancies. Of the 11 cases, 3 represented significant “undercalls” by the residents, in which the resident report was negative and the attending report indicated acute appendicitis (with perforation in 1 case). There was one significant “overcall” by a resident, in which the resident report described acute perforated appendicitis and the attending report indicated enteritis. The remaining seven discrepancies represented cases subjectively judged to be subtle, with six of the seven being cases initially called equivocal for acute appendicitis and with the attending report indicating no appendicitis. In one case, a negative resident read was reported as equivocal by the attending radiologist.

### Diagnostic performance

Residents and attending radiologists interpreted 90/377 (23.9%) and 92/377 (24.4%) examinations as positive for acute appendicitis, respectively, and the positive interpretation rate was not significantly different (*P*=0.53, McNemar’s test). Of the 90 positive interpretations by the residents, 83 were true positives and 7 were false positives. Of the remaining 287 negative interpretations by the residents, 279 were true negatives for acute appendicitis and 8 were false negatives. Of the 92 positive interpretations by the attending radiologists, 89 were true positives and 3 were false positives. Of the remaining 285 negative interpretations by the attending radiologists, 283 were true negatives for acute appendicitis and 2 were false negatives. Each attending false-positive and false-negative case was also reported as a false positive or false negative by the resident. These data are summarized in Fig. 1. The diagnostic performances of the attending radiologists and residents are summarized in Table 4.

**Table 4:** Diagnostic performances of attending radiologists and residents

Metric	Residents	Attending radiologists
Sensitivity	83/91, 91.2% (83.4–96.1%)	89/91, 97.8% (92.3–99.7%)
Specificity	279/286, 97.6% (95.0–99.0%)	283/286, 98.9% (97.0–99.8%)
Positive predictive value	83/90, 92.2% (84.6–96.8%)	89/92, 96.7% (90.8–99.3%)
Negative predictive value	279/287, 97.2% (94.6–98.8%)	283/285, 99.3% (97.5–99.9%)
Accuracy	362/377, 96.0% (93.5–97.8%)	372/377, 98.7% (96.9–99.6%)

Numbers in parentheses indicate 95% confidence intervals

### Alternative diagnoses

**Fig. 4:**
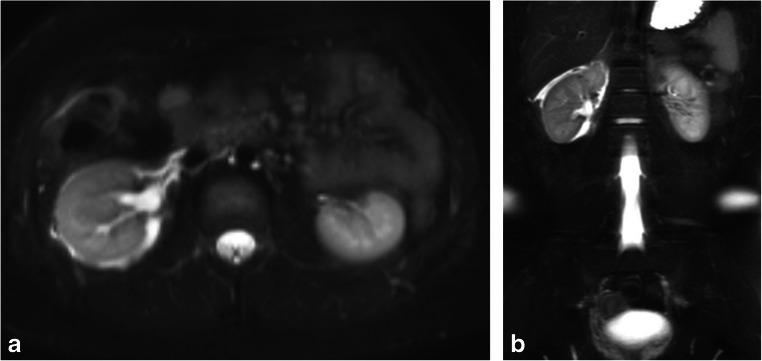
A 15-year-old boy with an alternative diagnosis of obstructing ureterolithiasis. **a**, **b** Axial (**a**) and coronal (**b**) fat-suppressed T2-weighted single-shot fast spin echo images demonstrate dilatation of the right renal collecting system and enlargement of the right kidney with respect to the left. There is right perinephric edema. This was a discrepant case in which the alternative diagnosis was missed by the interpreting resident. The appendix was normal

There were 93 cases in the data set that were negative for acute appendicitis and in which the final report indicated an alternate diagnosis that explained the patient’s abdominal pain. Common alternative diagnoses included enteritis/colitis, pyelonephritis and ovarian pathology; a more detailed description and tabulation of these alternative diagnoses can be found in the previous study [11]. The overall concordance rate for alternative diagnoses was 80.6% (75/93). A representative case is presented in Fig. 4.

## Discussion

This study demonstrates a high degree of concordance between resident and attending radiologist interpretations of abdominopelvic MRI performed in our emergency department for the evaluation of pediatric acute appendicitis. In addition, subjective analysis of the 11 cases of discrepant interpretations suggests that the majority of trainee errors occurred on studies that demonstrated subtle findings or were otherwise difficult to interpret.

There was no significant difference in the concordance rates when comparing residents by postgraduate year of training, suggesting that residents with as little as 1 year of radiology training before they started on-call responsibilities demonstrated diagnostic performance comparable to more experienced residents. At least one previous study has similarly shown no difference between resident postgraduate years [12], but the majority have demonstrated improvements in resident performance with increasing experience [13–18]. The high performance of junior residents in this study may be due, in part, to early focused training. Before undertaking overnight call, first-year residents at our institution undergo focused lectures and case reviews dedicated to familiarizing them with essential sequences, common pathologies and pitfalls. The residents have access to a teaching file of interesting cases to review. The first-year residents also undergo a mock pre-call test that includes emergency body MRI cases to assess their proficiency in interpreting these studies. Detailed educational and training approaches to interpreting MRI for pediatric acute appendicitis are available in the literature [19–21].

The accuracy of resident preliminary interpretations in emergent imaging studies has been widely investigated in the literature. Previous studies have shown that the concordance rates between resident and attending interpretations are generally high across a wide variety of modalities and clinical scenarios [12, 13, 15, 17, 22–24]. However, higher rates of discrepancy have been noted for specific modalities/diagnostic scenarios, including neuroradiology MRI [14] and CT angiography of the head/neck [16]. These studies suggest that concordance between residents and attending radiologists should be evaluated for specific modalities and diagnoses to identify scenarios in which trainees with limited experience may not perform as well.

Analysis of the diagnostic performance of both residents’ and attending radiologists’ interpretations with respect to “ground truth” showed that the differences were not statistically significant. However, trends in these data may indicate specific deficiencies that lead to discrepant interpretations. Resident sensitivity (91.2%) was lower than that for attending radiologists (97.8%) with overlapping 95% confidence intervals, suggesting that residents may miss subtle positive cases of acute appendicitis. Specificity was high for both groups. However, when applied to the study cohort (in which there was a relatively low rate of positive cases), resident positive predictive value (92.2%) trended lower in comparison to attending radiologists (96.7%) with overlapping 95% confidence intervals. Negative predictive value was high for both groups, but slightly lower for the residents. Considering the positive interpretation rates were similar (90/377, 23.9% for residents and 92/377, 24.4% for attending radiologists), in the context of our patient population, there appears to be a trend toward resident “overcalls,” rather than “undercalls.”

A particular benefit of MRI is its ability to suggest alternative causes for a patient’s abdominal pain. In this data set, 93 cases (24.7%) were found to demonstrate alternative diagnoses, most commonly in the form of enteritis/colitis, pyelonephritis and ovarian pathology. The overall rate of resident to attending radiologist concordance for these alternative diagnoses was lower than that for acute appendicitis, at 80.6%. Although these results are likely influenced by the nearly unlimited number of possible alternative diagnoses (as compared with the binary interpretation of positive or negative for acute appendicitis), they demonstrate additional benefit gained from expert interpretation of these examinations.

This study is limited by its retrospective nature. Additionally, images were not retrospectively evaluated, and the statistical analysis was performed using the radiology reports. We acknowledge that a retrospective image analysis wherein each MRI examination was evaluated by multiple residents of varying levels of training and attending radiologists would have resulted in a more robust scientific analysis; however, the aim of the study was to assess the diagnostic performance as reflected in a real-life scenario. The reference standards used in the study (histology and clinical follow-up) represent imperfect gold standards. In particular, clinical follow-up was unable to be standardized due to the retrospective nature of the study. Diagnostic performance for identifying features of complicated appendicitis was not examined and could be further investigated in future work. We did not compare MRI with other imaging modalities, specifically US.

## Conclusion

Abdominopelvic MRI for pediatric acute appendicitis can be accurately interpreted by trainees given appropriate targeted training, with performance comparable to that of attending radiologists. The modality is therefore likely appropriate for more widespread adoption outside of specialized tertiary care centers.
